# The Dynamics of Power Flow From the Global Health Financing: Comment on "Power Dynamics Among Health Professionals in Nigeria: A Case Study of the Global Fund Policy Process"

**DOI:** 10.34172/ijhpm.2023.7806

**Published:** 2023-05-10

**Authors:** Garrett Wallace Brown, Natalie Rhodes

**Affiliations:** ^1^University of Leeds, Leeds, UK; ^2^School of Politics and International Studies (POLIS), University of Leeds, Leeds, UK

**Keywords:** Power, Global Health Financing, Development Aid, Finance Colonialization, Epistemic Power

## Abstract

This article agrees with Lassa et al that biomedical paradigms and medical professionals are a dominating force within the policy dynamics of the Global Fund to Fight AIDS, Tuberculosis and Malaria (GFATM) and that there needs to be greater community involvement in how global health initiatives (GHIs) are adopted, designed, implemented and evaluated. However, we argue that many of the conditions identified are entrenched and perpetuated by how GHIs are financed and the financing modalities employed in Development Aid for Health (DAH), particularly in low resource settings. As a result, the dynamics of power not only flow from traditionally entrenched epistemic authorities but are disproportionally sustained by global health financing modalities that favour particular GHIs over others. As we argue, these DAH modalities can exert forms of power with problematic effects on policy-making.

## The Importance of Understanding Power in Global Health

 Lassa et al^1^ identified four phenomena when examining dynamics of power within the policy process of the Global Fund to Fight AIDS, Tuberculosis and Malaria (GFATM) in Nigeria. First, they argue that medical professionals dominated the policy landscape and thus had disproportional influence on how policy is shaped and implemented. Second, this influence resulted in a greater focus on clinical, biomedical and supply-side interventions, largely at the exclusion of demand-side programs. Third, the dominance of medical professionals came at the cost of wider community engagement and representation, which was stated to be a particular problem within the make-up of the Country Coordination Mechanism (CCM), a key GFATM multisectoral instrument. Lastly, the lack of community input was stated to underrepresent communal public health needs and the identification of contextual factors that moderate the effectiveness, efficiency and equity of GFATM global health initiatives (GHIs). In the case of the latter, this threatened to produce underperforming GFATM programs that can ultimately undermine positive public health outcomes.

 Highlighting these dynamics of power is hugely important and enables us to critically appraise multisectoral and community involvement within GFATM policy processes so as to identify reforms for better community representation, procedural legitimacy, and the addressing of demand-side issues. As argued by Lassa et al, in the case of Nigeria, disproportional influence within GFATM processes can result in skewed policy foci and modes of operationalisation, requiring greater community involvement in determining what GHIs are adopted and how they are designed, implemented and evaluated.

## The Same as It Ever Was: Biomedical Paradigms Rule the Roost

 In everyday practice the dynamics outlined by Lassa et al are prevalent in global health policy more broadly and the disproportional influence of medical professionals, particularly medical doctors, on public health decision-making is well entrenched. When surveying leadership of public health institutions in most countries (whether it is high-income, low- and middle-income or low-income countries) it is often over-represented by former medical doctors or those formally trained as medical doctors. Even where there is a greater representation of individuals with a Master of Public Health or other health related degrees within a policy-making process, there is often deference toward medically trained doctors due to longstanding epistemic hierarchies.

 Moreover, due to how health professionals are trained, it is not surprising that Western medicine and its ‘best practices’ remain hegemon and that this shapes how health professionals’ approach public health. This criticism is not new and is constantly stressed within debates about traditional and complimentary medicine.^2,3^ Thus, it is reasonable to argue that there is an inherent bias for Western styled clinically based supply-side GHIs that mirror protocols often refined by Anglo-European institutions.^4^ For better or worse, these biases can affect which GHIs are chosen and how they are designed, implemented, monitored and evaluated.

 Lastly, there has been an increased strengthening of the biomedicalization of health as part of an intensified focus on global health security — a condition that has accelerated in response to COVID-19 and within emerging pandemic preparedness and response agendas.^5,6^ As recently argued by Holst and van de Pas,^7^ when surveying the literature on health security, there is ‘a trend towards biomedical solutions’ which often ‘neglects root causes of global health crises.’ What this biomedicalization and securitization of health renders is an implicit bias toward surveillance, field epidemiology, diagnostics, containment, border control, and medical and pharmaceutical countermeasures. Resultingly, this bias largely ignores wider health system strengthening needs, corresponding human resources, and other preventative measures aimed at upstream determinants.^6^

 Nevertheless, it would be churlish to look upon the role of health professionals, health security and formal biomedical training in purely negative terms. This is for two reasons. First, health professionals, particularly local personnel, have the necessary first-hand experience and knowledge required for understanding health system needs and health security risks. Although communities will also have very useful experience and knowledge, it is complimentary knowledge, not a substitute. Second, the rise of input from health professionals within GHIs is a progressive step forward. For example, in the early 2000s, during debates about building better ‘global health partnerships,’ the argument was to get more ‘front-line’ health professionals into the policy-making process and to decentralise decisions to local level implementors.^8,9^ The upside was that this allowed more district and facility level knowledge to filter into the policy process, which has arguably helped to create more context specific programs. And this was the logic and aim underwriting the GFATM CCM model.^10^ Yet, this has now given managing doctors and health professionals lopsided access to how local and national level health policy interfaces with global level GHIs. Thus, although the focus has now shifted to the need to increase community engagement and inclusion, we should also not forget the increased value of having more health professionals involved.

 What this suggests is that a better balance is required. One where CCMs represent a larger range of voices and stakeholders. Yet, this is not easily done. For example, early CCM evaluations determined that less than half of them met the multisectoral threshold recommended by the GFATM.^10^ Moreover, the selection process for CCM membership is often difficult to ascertain and can be directed by deep-rooted interests within departments of public health and national health ministries.^10^ Depending on CCM leadership there can be more inclusion or exclusion of wider community voices, while in some cases, CCM chairs can promote cronies and outside interests.^11^ Furthermore, CCMs tend to be chaired and managed by medical doctors or those trained in specifically Western styled public health.^11^ Thus, it is not surprising that key ideational properties remain ‘built into’ the CCM policy space and their interlocutions with the GFATM. Lastly, CCM engagement and inclusion activities often have limited budgets which operate within already constrained health systems. This can undermine wider involvement, particularly people from low resource and remote communities.^10^ This does not excuse the GFATM and national governments for not doing more to address these shortcomings, but it does help to explain the challenges policy-makers face in doing so, while also giving more insight into the key concerns outlined by Lassa et al.

## Financial Nudging and Incentivization Perpetuates the Paradigm

 Although entrenched epistemic paradigms and professional hierarchies are clear levers of power within GHI decision-making, this condition is actually entrenched and perpetuated by how GHIs are financed and incentivized, particularly in low resource settings. As a result, the dynamics of power not only flow from traditionally established epistemic authorities but are disproportionally driven by global health financing modalities that favour particular GHIs over others.^6^ In many ways this sets the foundation for who, what and how GHIs are created, thus engraining paradigms, inclusion criteria, and policy parameters. Below we give just two examples of this effect relevant to the GFATM.

 As noted by Lassa et al, the Technical Review Panel (TRP) reviews and recommends all GFATM grant proposals. This procedural hurdle can be understood to fulfil two functions. First, it is a procedure for helping to better determine whether a proposal reflects ‘best practice’ as well as its ‘feasibility.’ Here, the TRP measures grant proposals against traditional biomedical standards within existing paradigms. Although the TRP has gone through a number of reforms to become less ‘Western centric’ in response to past criticism, there are arguments that the process is not ‘decolonialized’ enough. Second, and most importantly, the role of the TRP procedure is to make sure that the grant will maximize ‘value for money’ and ‘accountability’ to donors. This engrains a certain structure-agent power dynamic that usually flows one way while fixing these conditionalities into financial flows.^12^ In this regard, the TRP is lockstep with Development Aid for Health (DAH) performance management in general. And similar accountability mechanisms are repeated in most global institutions through a number of results-based financing modalities.

 Although demand-side initiatives are more likely to be found within GFATM grants (eg, Soul city in South Africa - Round One), there is a tendency in DAH to shy away from them. The justification for this is often made explicit by donors, which greatly influences national grant design.^13^ For example, donors and their institutional proxies often argue that demand-side initiatives are hard to quantify and track. Whereas supply-side initiatives, say like immunizations, can more easily allow for the tracking of each discernible unit purchased as well as the corresponding number of jabs given. Moreover, it is often argued that supply-side interventions are more compatible with existing monitoring and evaluation systems, allowing for easier implementation and complementarity. It is furthered argued that this allows greater assurance of data being reported, which in turn, allows better analysis. In contrast, demand-side programmes like increased facility transportation in remote areas, incentives for increasing patient facility visits, and outreach programs are more difficult to monitor and evaluate. In much simpler terms, demand-side initiatives are much harder to count and be accounted for. As a result, there is an inherent bias within the global health financing landscape, and donors tend to prefer supply-side interventions that allow easy tracking and accountability. This logic helps to explain why GHIs tend to be vertical and siloed. Moreover, it explains why some global initiatives explicitly exclude demand-side interventions as part of their performance-based financing schemes, as is the case with the World Bank’s Global Financial Facility.^14^

 Nevertheless, a key downside of ignoring demand-side programs is that this can undermine efforts to increase access by addressing contextual moderators such as poor transportation, remoteness, poor health communication, low incentives for maintaining regular preventative health, and general community support.^14^ As a result, maintaining a supply-side focus will remain necessary, but insufficient, since it ignores important policies aimed at increasing community engagement and addressing social determinants of health.^15^ This can translate into less effective, efficient and equitable GFATM interventions, since these diseases thrive in impoverished areas where low education and social opportunities make long-term disease management challenging.

## Money Is Power and Much Will Follow

 Although the importance of community and civil society engagement is frequently lauded, the degree of meaningful engagement varies across GHIs.^16^ As demonstrated by Lassa et al, even with formal engagement mechanisms in place, there is no guarantee of fair representation across relevant stakeholders. This is unfortunate, since the inclusion and representation of diverse stakeholders beyond governmental or societal elite is a key aspect in reducing democracy deficits within decision-making processes, particularly for decisions intended to be for the public good.^12^ Yet, as evidenced by Lassa et al, such engagement mechanisms alone cannot overcome obstacles posed by power asymmetries and as such permit epistemic injustices to continue. Therefore, even when the representation of stakeholders is more equal, this does not always equate to equal influence. In the case of global health policy, and its financiers, the ‘truth-holder’ continues to be well-resourced Global North actors, who typically assume a technocratic biomedical view of health.^7,17-19^

 As suggested earlier these power differentials manifest first at the global level. For instance, it is often the case that international non-governmental organizations (NGOs) with greater resources and connections are appointed positions as civil society representatives in place of indigenous NGOs.^20,21^ Donors also often require projects to be implemented by appointed international consultants with local experts, where community level stakeholders are often relegated to output and reporting duties, thus side-lining local expertise and knowledge.^22^ Additionally, it often appears that widening engagement and participation in GHIs, particularly for civil society actors, remains an afterthought. For example, the newest global health financing instrument is the Pandemic Fund announced by the World Bank in early 2022. Whilst civil society representatives have now been given two voting seats on the governing board, this space was only created in the final stages of the design process and only after considerable lobbying. Similarly, for the Access to COVID-19 Tools Accelerator, civil society representation was included in the governance structure only after the initiative was well underway.^23^ In both cases these GHIs were largely donor driven and subject to their financing considerations, with its cascading effect on ‘implementing countries.’

 Consequently, we argue that a key driver of these power dynamics is the desire of donors to retain as much control and oversight over their funds and financing models as possible. Recognizing this helps to explain the donor preference for earmarked funding and vertical programs as well as the bias often given to supply-side clinical and biomedical projects. In both cases, these allow for neat measurable indicators. Finally, it helps explain the dominance of international NGOs and clinical experts, who are more attuned and better trained in donor mindsets, granting them greater voice as well as DAH capture. Whilst simplistic, it is this dynamic which then reduces the space for inclusion of more community-led participation. Ultimately side-lining local expertise and community perspectives.

## Ethical issues

 Not applicable.

## Competing interests

 Authors declare that they have no competing interests.
